# Indoor cycling training in rehabilitation of patients after myocardial infarction

**DOI:** 10.1186/s13102-021-00379-w

**Published:** 2021-11-29

**Authors:** Dagmara Gloc, Zbigniew Nowak, Agata Nowak-Lis, Tomasz Gabryś, Urszula Szmatlan-Gabrys, Peter Valach, Anna Pilis

**Affiliations:** 1 Silesian Center for Rehabilitation and Prevention, 43-450 Ustron, Poland; 2 Department of Physiotherapy, Jerzy Kukuczka Academy of Physical Education, 40-065 Katowice, Poland; 3 Sport Centrum Faculty of Pedagogy, University of West Bohemia, 301 00 Pilsen, Czech Republic; 4 Department of Anatomy, Faculty of Rehabilitation, University of Physical Education, 31-571 Kraków, Poland; 5 Faculty of Health Science, Jan Dlugosz University, 42-200 Czestochowa, Poland

**Keywords:** Myocardial infarction, Cardiac rehabilitation, Indoor cycling

## Abstract

**Background:**

Standard endurance training used from the second stage of cardiac rehabilitation has many common features with indoor cycling training which is used in fitness clubs. In the study, an attempt was made to evaluate the usefulness of this form of training in a 24-day rehabilitation program for patients after myocardial infarction. The study examined a group of 64 patients (51.34 ± 8.02 years) who were divided into two groups: the IC group (32 patients aged 53.40 ± 4.31 years) with indoor cycling training instead of standard endurance training; and the ST group (32 patients aged 55.31 ± 6.45 years) performing standard training. The level of exercise tolerance (cardiopulmonary exercise testing on a treadmill—Bruce’s protocol), hemodynamic indicators of the left ventricle (echocardiography) and blood lipid profile (laboratory test) were assessed.

**Results:**

In the IC group there was a significant increase in the test duration (9.21 ± 2.02 vs 11.24 ± 1.26 min; *p* < 0.001), the MET value (9.16 ± 1.30 vs 10.73 ± 1.23; *p* = 0.006) and VO_2_max (37.27 ± 3.23 vs 39.10 ± 3.17 ml/kg/min; *p* < 0.001). Parallel changes were observed in the ST group, where the following parameters improved: the test duration (9.41 ± 0.39 vs 10.91 ± 2.22; *p* < 0.001), MET value (8.65 ± 0.25 vs 9.86 ± 1.12; *p* = 0.002) and VO_2_max (36.89 ± 6.22 vs 38.76 ± 3.44; *p* < 0.001). No statistically significant changes were found in the hemodynamic indices of the left ventricle and the lipid profile. Also, the intergroup analysis did not show any statistical significance.

**Conclusion:**

Based on the research results, it was found that indoor cycling training in the second phase of cardiac rehabilitation is a safe form of therapy and therefore may be an interesting alternative method to the classic bicycle ergometer exercise in the stage of early cardiac rehabilitation.

## Introduction

Traditional physical training, in line with the existing standards of management, which begins in the second stage of cardiac rehabilitation, is well known and described [1–3]. Few publications deal with the issue of the effectiveness of innovative forms of training in patients with diseases of the cardiovascular system [4–6]. The ever younger age of cardiac patients, the progress of diagnostics and the development of interventional and surgical treatment methods require the gradual introduction of various forms of physical training. Adapting the rehabilitation programs to the possessed skills based on previous experience in the field of activity is becoming a key solution in the selection of training programs in modern cardiac rehabilitation. One such form is indoor cycling [7–9]. It is a form of aerobic interval training similar to the traditional training recommended in the second stage of rehabilitation, but with a slightly different course. Participants are not divided into beginners and advanced, because each person during the course of classes can modify loads or positions according to their own abilities [10, 11]. The training imitates cycling in two basic types of terrain—flat and hilly. Riding can take place with and without contact of the arms with the steering wheel, in a sitting and standing position. It is already possible to change the position of the hands (1. Close, 2. Open, 3.Standing), allowing a variety of rehabilitation activities. The techniques used in indoor cycling reflect road cycling or in professional cycling sports and are divided into: seated flat (SF) cadence 80–120 rpm, seated climb (SC) cadence 60–80 rpm, standing flat (StF) cadence 80–120 rpm, standing climbing (StC) cadence 60–80 rpm), combo (Co) cadence 60–80–120 rpm, sprint flat (SpF) and sprint climb (SpC) cadence, maximum 100–110 rpm. The presented diversity of solutions used during exercise on a bicycle creates the possibility of conducting frequently changing classes in terms of exercise technique. An additional attraction of such training is the use of music, which makes it possible to determine the intensity of the traveled section, as well as its profile. The most frequently used music genres for classes are pop, techno, hip-hop, disco, and reggae.

The purpose of the class determines the profile, which can be:

- “active regeneration”/pro-health training (50–65% HRmax),
- “endurance low/fat burning” (65–75% HRmax)
- “endurance” (75–90% HRmax)
- “climbing” (75–85% HRmax),
- “interval” (65–85% HRmax)
- “challenge” (85–100% HRmax)
- “first time/express” (65–75% HRmax) [7.8].

Scientific reports confirm the effective impact of indoor cycling training on cardiovascular and respiratory efficiency, reduction of adipose tissue and the risk of developing cardiovascular diseases in people without cardiovascular diseases [12–17]. The research conducted so far indicates the validity of undertaking research aimed at assessing the use of indoor cycling training as an alternative form to traditional endurance training in the primary and secondary prevention of cardiovascular diseases.

Due to the lack of reports on the possibilities of using indoor cycling training in cardiac rehabilitation programs, an experiment was conducted to determine the effect of such training on the level of exercise tolerance and hemodynamic indices of the left ventricle in patients after a heart attack.

The following research questions were formulated:

1. Can indoor cycling training, like standard endurance training, improve exercise tolerance (assessed by an exercise test) and change hemodynamic indicators of the left ventricle (assessed by echocardiography) and lipid profile (assessed using a laboratory method) in patients after myocardial infarction?
2. Can indoor cycling training be an alternative to standard endurance training commonly used in stage II rehabilitation?

### Hypotheses

- Indoor cycling training may improve exercise tolerance, left ventricular hemodynamic indices and the lipid profile of patients after a heart attack.
- Indoor cycling training can be an alternative to standard endurance training used during the second stage of cardiac rehabilitation.

## Material and methods

### Participants

The study included 64 men (51.34 ± 8.02 years), after myocardial infarction, who underwent percutaneous coronary angioplasty. The tests were performed during the second stage of rehabilitation. All participants of the experiment were qualified for model A (exercise test result ≥ 7 MET or 100 W). Reducing the number of confounding factors, such as age, sex, disease entity, treatment method, and level of exercise tolerance, patients included in the study were randomized to two rehabilitation procedures:

- standard rehabilitation (group ST)—32 people
- indoor cycling training (group IC)—32 people

*Inclusion criteria* consent to participate in the study, documented stable ischemic heart disease or an uncomplicated course of myocardial infarction, time from the last cardiovascular event < 2 months, stress test result ≥ 7 MET/100 W, left ventricular ejection fraction (LVEF) ≥ 50%. Exclusion criteria: refusal to participate in the study, recent myocardial infarction, LVEF < 50%, coronary artery bypass surgery, unregulated hypertension, unstable coronary artery disease, arrhythmias and conduction disturbances, established cancer, diseases of the central or peripheral nervous system, varicose veins of the lower limbs, osteoarthritis of the peripheral joints and the spine, unhealed injuries of the lower limbs, advanced peripheral arteriosclerosis, age ≥ 75 and incomplete medical documentation.

### Experimental procedure

Both the standard group and the IC group were subjected to a 24-day improvement program, which included 22 training units (2 days for initial and final tests) performed 5 times a week following ESC standards (a detailed training program is presented in Table 1. Throughout the entire research procedure, the patients were supervised by medical personnel consisting of a physiotherapist and a cardiologist.

**Table 1:** Training following ESC recommendations

Type of training	Methodology	Workload
Endurance training	Training on a bicycle ergometer, 5 times a week 30 min	Workload applied on the basis of calculation of heart rate training, starting from 60% of heart rate reserve increased by 10% after 5 units of training, to 80% of heart rate reserve, 14 degrees of subjective scale effort assessment by the Borg scale
Resistance training	Exercises in the form of training station, 5 times a week 30 min	
General exercises	Exercises in the gym-elements of aerobic and anaerobic training, stretching, breathing exercises, 5 times a week 30 min	

The intensity of the exercise varied on the basis of the calculated training heart rate, starting from 60% of the heart rate reserve, increasing by 10% after 5 training units, up to 80% of the heart rate reserve, up to a maximum of 15 according to the Borg scale.

Endurance training on a bicycle ergometer (Kettler Ergometer X1) began with a 3-min ride without load (0 W), followed by 5 cycles—a 3-min load phase and a 2-min rest phase, a total of 25 min of riding. The training session ended with 2 min of cycling without load. The training on a bicycle ergometer lasted a total of 30 min. After each training unit, stretching of the muscle groups involved during the ride was performed (5 min). People from the IC group performed indoor cycling training (Tomahawk I.C.E. Indoor Cycling) instead of the traditional interval training. The training ride lasted 30 min in total and started with a 5-min warm-up with no load, in the rhythm of 100–110 RPM. The main part included cycling to the rhythm of changing music, including sitting and standing positions, and lasted 22.5 min (60–110 RPM). The main part was followed by a 2.5-min cool down with a gradually decreasing load. The cycling cadence (RPM) was determined by the rhythm beats (BPM) present in each piece, being an important motivating instrument; moreover, the cadence of the rotation was signaled by the instructor. The unit ended with 5 min of stretching of the muscle groups—the muscles of the chest, back, quadriceps and biceps muscles of the thigh, as well as the buttocks, forearms and arms. The stretching was performed on mats placed on the dance floor. Details of the training protocol are presented in Table 2. Patients from the experimental group also participated in the other two forms of training (resistance and general improvement), as did the control group (Table 1).

**Table 2:** Protocol of the indoor cycling training unit

Part of the training session	Time (min)	Borg scale	RPM	Position/technique
Warm-up	1–5	9–10	100–110	Position 2 (2½min) Position 1—SF (2½min)
Appropriate training	5–10	12–13	110	Position ja 1 (2 min)
			110	Position a 2 (2 min)
			80	Position a 2—SC (1 min)
	10–17.5	12–14	80	Position a 3—StC (½min)
			100–110	Position a 1 (2½min)
			100	Position a 2 (2½min)
			60–80	Position a 3 StC (½min)
			100	Position 1 (1½min)
	17.5–22.5	13–14	60–80	Position ja 2—SC (1 min)
			100	Position 2 (3½min)
			80	Position 3—StC (½min)
	22.5–27.5	11–12	80	Position 2 (2 min)
			60–80	Position a 2—SC (2 min)
			100–110	Position a 2 (1 min)
Cool down	27.5–30	9–10	100	Position a 1—SF
Stretching	30–35	9	–	–

*min* minute, *position1* close, *position 2* open, *position 3* standing, *RPM* revolutions per minute, *SC* seated climb, *SF* seated flat, *StC* standing climb, *StF* standing flat

The following was carried out before commencing the training program and immediately after its completion:

- Electrocardiographic exercise test on a treadmill (six-stage Bruce protocol: stage 1 = 2.7 km/h, 10%, stage 2 = 4.0 km/h, 12%, stage 3 = 5.5 km/h, 14%, stage 4 = 6.8 km/h, 16%, stage 5 = 8.0 km/h, 18%, stage 6 = 8.8 km/h, 20%) Exercise test using the Excalibur Sport cycle ergometer (Lode, Groningen, The Netherlands) [18]. The following were measured: test duration (min), distance covered (m), energy cost (MET), heart rate at rest and maximum (BPM), systemic blood pressure at rest and maximum (mmHg), criteria for ending the test (physiological: submaximum heart rate, i.e., 85% of HRmax determined on the basis of the following formula: 208–0.7 × age or fatigue; pathological: stenocardial pain, ST segment, and T-wave changes, rhythm and/or conduction disorders, blood pressure increase above 250/120 mmHg), maximum oxygen uptake (VO_2_max).
- Two-dimensional ultrasound heart test, measured hemodynamic parameters (GE Vivid Q): left ventricular end-diastolic dimension (LVEDD; mm), left ventricular end-systolic dimension (LVESD; mm), left ventricular end-systolic volume (LVESV; mL) as per the following formula:

$${\text{LVESV }} = {\text{ 7}}/\left( {{\text{2}}.{\text{4 }} + {\text{ LVESD}}} \right) - {\text{ }}\left( {{\text{LVESD}}} \right),{\text{ left ventricular end}} - {\text{diastolic volume }}\left( {{\text{LVEDV}};{\text{ mL}}} \right)$$

as per the following formula: LVEDV = 7/(2.4 + LVEDD) − (LVEDD), left ventricular ejection fraction (LVEF; %), left ventricular mass (LVM; g), left ventricular mass index (LVMI; g/m^2^) based on the Devereux formula: LVMI = LVM/BSA (left ventricular mass/body surface area).

- Blood lipid profile test. Measured parameters:

Total cholesterol—TC (mg/dL), high-density lipoproteins—HDL (mg/dL), low-density lipoproteins—LDL (mg/dL), triglycerides—TG (mg/dL).

Before, during and immediately after each training session, heart rate (Polar, FT1) and blood pressure (SOHO, 110 HS-50A) measurements were made, as well as the degree of perception of effort according to the 20-point Borg scale. The intensity of the exercise varied on the basis of the calculated training heart rate, starting from 60% of the heart rate reserve, increasing by 10% after 5 training units, up to 80% of the heart rate reserve, up to a maximum of 15 according to the Borg scale.

The following was carried out before commencing the training program and immediately after its completion:

- Electrocardiographic exercise test on a treadmill (six-stage Bruce protocol: stage 1 = 2.7 km/h, 10%, stage 2 = 4.0 km/h, 12%, stage 3 = 5.5 km/h, 14%, stage 4 = 6.8 km/h, 16%, stage 5 = 8.0 km/h, 18%, stage 6 = 8.8 km/h, 20%) Exercise test using the Excalibur Sport cycle ergometer (Lode, Groningen, The Netherlands) [18]. The following were measured: test duration (min), distance covered (m), energy cost (MET), heart rate at rest and maximum (BPM), systemic blood pressure at rest and maximum (mmHg), criteria for ending the test (physiological: submaximum heart rate, i.e., 85% of HRmax determined on the basis of the following formula 208–0.7x age or fatigue; pathological: stenocardial pain, ST segment, and T-wave changes, rhythm and/or conduction disorders, blood pressure increase above 250/120 mmHg), VO_2_max.
- Two-dimensional ultrasound heart test, measured hemodynamic parameters (GE Vivid Q): LVEDD (mm), LVESD (mm), LVESV (mL) as per the following formula:
  $${\text{LVESV }} = { 7}/\left( {{2}.{4 } + {\text{ LVESD}}} \right) \, - \, \left( {{\text{LVESD}}} \right),{\text{ LVEDV }}\left( {{\text{mL}}} \right)$$

as per the following formula: LVEDV = 7/(2.4 + LVEDD) − (LVEDD), LVEF (%), left ventricular mass (LVM; g), left ventricular mass index (LVMI; g/m^2^) based on the Devereux formula: LVMI = LVM/BSA (left ventricular mass/body surface area).

- Blood lipid test profile. Measured parameters:

total cholesterol—TC (mg/dL), high-density lipoproteins—HDL (mg/dL), low-density lipoproteins—LDL (mg/dL), triglycerides—TG (mg/dL) [18]

### Data analysis

The Shapiro–Wilk normality test and the Brown–Forsythe variance homogeneity test were used to verify the assumptions of parametric tests. The parametric Student’s *t* test was also performed for dependent variables whose distribution conformed to a normal distribution, and a nonparametric Wilcoxon paired order test was performed for dependent variables whose distribution did not conform to a normal distribution. Student’s t-test was also performed for independent variables whose distribution conformed to a normal distribution, and its nonparametric equivalent. The Mann–Whitney U-test was performed for independent variables whose distribution did not conform to a normal distribution. Statistica 12 (StatSoft, Kraków, Poland) software was used in the study. The assumed level of significance was *p* _ 0.05.

## Results

The characteristics of the subject are presented in the Tables 3, 4 and 5.

**Table 3:** Characteristics of both groups

Variable	Group IC (N = 32)	Group ST (N = 32)
Age (years)	53.40 ± 4.31 (38–74)	55.31 ± 6.45 (41–72)
Height (cm)	177 ± 4.66 (169–189)	178.20 ± 7.55 (163–188)
Weight (kg)	85.65 ± 7.55 (72–101)	86.09 ± 13.22 (66.90–111.24)
BMI (kg/m^2^)	26.48 ± 1.61 (22.28–33.42)	27.82 ± 6.63 (22.80–31.23)
LVEF (%)	55.22 ± 5.95 (51–59)	54.50 ± 8.44 (52–58)

*BMI* body mass index, *N* number, *LVEF* left ventricular ejection fraction

**Table 4:** Disease entities occurring in patients of both groups

Type	Group IC N (%)	Group ST N (%)
Ischemic heart disease	27 (84.3%)	28 (87.5%)
Type 2 diabetes	6 (18.75%)	8 (25%)
Hyperlipidemia	9 (28.12%)	11 (34.37%)
Hypertension	14 (43.75%)	18 (56.25%)
Myocardial infarction	32 (100%)	32 (100%)

**Table 5:** Type of myocardial infarction

Type	Group IC N (%)	Group ST N (%)
NSTEMI	26 (81.25%)	28 (87.5%)
STEMI	6 (18.75%)	4 (12.5%)
Total	32 (100%)	32 (100%)

*NSTEMI* non-ST elevation myocardial infarction, *STEMI* ST elevation myocardial infarction

Ischemic disease and myocardial infarction were dominant in both groups (Table 6).

**Table 6:** Number of implanted stents

Number	Group IC N (%)	Group ST N (%)
1	27 (84.37%)	28 (87.5%)
2	4 (12.51%)	4 (12.5%)
≥ 3	1 (3.12%)	0 (0%)

Implantation of 1 stent was predominant in all groups.

Table 7 shows the results for the two test groups of the treadmill exercise test as per the classical Bruce protocol. In both analyzed groups, after the completion of the rehabilitation programs, a statistically significant increase in test duration, energy cost (MET) and VO_2_max was demonstrated in relation to the baseline test.

**Table 7:** Results of the treadmill exercise test in three groups of patients before (I) and at the end (II) of cardiac rehabilitation

Variable	IC group X ± SD	*p*	ST group X ± SD	*p*	Δ IC versus Δ ST *p* value
Time I	9.21 ± 2.02	< 0.001	9.41 ± 0.39	< 0.001	0.772
Time II	11.24 ± 1.26		10.91 ± 2.22		
Δ (min)	2.03		1.50		
MET I	9.16 ± 1.30	0.006	8.65 ± 0.25	0.002	0.873
MET II	10.73 ± 1.23		9.86 ± 1.12		
Δ MET	1.57		1.21		
HRrest I	66.32 ± 9.16	0.862	70.11 ± 8.15	0.790	0.899
HRrest II	65.30 ± 10.31		68.40 ± 7.71		
Δ (beats/minute)	− 1.02		− 1.71		
HRmax I	128.25 ± 14.79	0.169	128.55 ± 14.69	0.264	0.992
HRmax II	133.65 ± 16.37		133.75 ± 11.10		
Δ (beats/minute)	5.4		5.2		
SBPrest I	124.60 ± 11.59	0.999	124.40 ± 13.96	0.936	0.914
SBPrest II	125.45 ± 18.11		123.88 ± 2.38		
Δ (mmHg)	0.85		− 0.52		
DBPrest I	82.10 ± 6.52	0.984	79.25 ± 4.16	0.918	0.918
DBPrest II	81.05 ± 7.26		79.34 ± 6.27		
Δ (mmHg)	− 1.05		0.09		
SBPmax I	169.25 ± 11.30	0.663	158.50 ± 10.15	0.730	0.114
SBPmax II	174.09 ± 12.25		162.33 ± 13.53		
Δ (mmHg)	4.84		3.83		
DBPmax I	84.21 ± 6.48	0.954	83.85 ± 6.50	0.916	0.922
DBPmax II	82.20 ± 9.33		82.09 ± 6.18		
Δ (mmHg)	− 2.01		− 1.76		
VO_2_max I	37.27 ± 3.23	< 0.001	36.89 ± 6.22	< 0.001	0.167
VO_2_max II	39.10 ± 3.17		38.76 ± 3.44		
Δ (ml/kg/min)	1.83		1.87		

All data are presented as means ± standard deviation and the difference (Δ – delta)

*MET* metabolic equivalent, *HRrest* resting heart rate, *HRmax* maximum heart rate, *DBPmax* maximum diastolic blood pressure, *DBPrest* resting diastolic blood pressure, *SBPmax* maximum systolic blood pressure, *SBPrest* resting systolic blood pressure, *VO*_*2*_*max* maximal oxygen uptake

The results of the echocardiographic examination are presented in Table 8. In both studied groups, improvement in the analyzed hemodynamic parameters of the left ventricle, however, were not statistically significant. There was also no significant difference in intergroup comparisons.

**Table 8:** Results of echocardiographic tests carried out before (I) and after (II) the 24-day rehabilitation cycle

Variable	IC group X ± SD	*p*	ST group X ± SD	*p*	Δ IC versus Δ ST *p* value
LVEDD I	49.98 ± 3.60	0.918	50.33 ± 4.12	0.843	0.825
LVEDD II	50.21 ± 3.34		50.95 ± 3.23		
Δ (mm)	0.23		0.62		
LVESD I	32.71 ± 4.64	0.933	33.22 ± 3.11	0.773	0.818
LVESD II	32.89 ± 7.83		33.75 ± 3.28		
Δ (mm)	0.18		0.53		
LVESV I	44.33 ± 21.34	0.932	47.22 ± 11.21	0.892	0.899
LVESV II	45.11 ± 16.97		48.01 ± 10.01		
Δ (ml)	0.78		0.79		
LVEDV I	116.32 ± 22.52	0.911	121.12 ± 23.22	0.902	0.948
LVEDV II	117.44 ± 14.66		122.43 ± 14.38		
Δ (ml)	1.12		1.31		
LVSV I	84.12 ± 27.60	0.991	94.92 ± 31.43	0.981	0.932
LVSV II	84.01 ± 21.11		94.12 ± 13.34		
Δ (ml)	− 0.11		− 0.8		
LVEF I	55.22 ± 5.95	0.117	54.50 ± 8.44	0.197	0.911
LVEF II	56.45 ± 2.68		55.75 ± 9.44		
Δ (%)	1.23		1.25		
LVM I	180.56 ± 81.11	0.998	191.43 ± 19.33	0.935	0.989
LVM II	181.34 ± 22.46		192.19 ± 23.91		
Δ (g)	0.78		0.76		
LVMI I	89.64 ± 22.45	0.917	95.47 ± 16.75	0.933	0.886
LVMI II	90.33 ± 14.36		96.09 ± 54.64		
Δ (g/m^2^)	0.69		0.62		

*LVEDD* left ventricular end-diastolic diameter, *LVESD* left ventricular end-systolic diameter, *LVESV* left ventricular end-systolic volume, *LVEDV* left ventricular end-diastolic volume, *LVSV* left ventricular stroke volume, *LVEF* left ventricular ejection fraction, *LVM* left ventricular mass, *LVMI* left ventricular mass index

The analysis of changes in the lipid profile (Table 9) showed a favorable direction of changes in the values of all assessed indicators. However, these changes did not show statistically significant features.

**Table 9:** Results of blood lipid profile tests carried out before (I) and after (II) the 24-day rehabilitation cycle

Variable	IC group X ± SD	*p*	ST group X ± SD	*p*	Δ IC versus Δ ST *p* value
TC I	182.24 ± 26.41	0.249	172.27 ± 45.34	0.113	0.601
TC II	171.67 ± 10.39		166.23 ± 66.29		
Δ (mg/dl)	− 10.57		− 6.04		
HDL I	44.21 ± 19.07	0.142	43.23 ± 24.56	0.158	0.893
HDL II	48.34 ± 19.27		47.88 ± 17.66		
Δ (mg/dl)	4.13		4.65		
LDL I	111.23 ± 19.44	0.223	104.39 ± 24.34	0.223	0.367
LDL II	99.62 ± 26.12		91.47 ± 13.04		
Δ (mg/dl)	− 11.61		− 12.92		
TG I	121.35 ± 71.37	0.815	126.22 ± 21.56	0.793	0.829
TG II	115.15 ± 45.32		119.48 ± 14.59		
Δ (mg/dl)	− 6.2		− 6.74		

*TC* total cholesterol, *HDL* high-density lipoproteins, *LDL* low-density lipoproteins, *TG* triglycerides

## Discussion

There are a small number of publications on the impact of alternative forms of endurance training used in the second stage of rehabilitation on the level of exercise tolerance, hemodynamic parameters of the left ventricle, or the lipid profile of patients after a myocardial infarction [4, 6].

Never before has such an assessment been made of indoor cycling training.

Until now, this form was available and associated only with the population of healthy people who attended classes in fitness clubs. It has many features in common with traditional endurance training that has been used for years in a cardiac rehabilitation program. These include: the interval training form, HR-controlled work intensity, the ability to control and dose external resistance, individual or group training form, constant monitoring of vital signs (HR, SpO_2_, blood pressure, Bf), low risk of injury. What makes it stand out is primarily the way it is run. It is possible to ride sitting and standing, as well as to adjust the height of the saddle and handlebars and the distance between the saddle and the handlebars, which makes it an ideal training device for people regardless of their constitutional body build [7–9]. The results obtained after the end of the cardiac rehabilitation program, in which indoor cycling was used, showed that it is a safe, effective and well-tolerated form of endurance exercise, which can be recommended in the process of comprehensive rehabilitation of patients after a heart attack.

### Electrocardiographic exercise test

The results obtained after 24 days of implementation of the rehabilitation program showed a significant improvement in physical capacity compared to the results obtained before its commencement. In both analyzed groups, i.e. the IC group and the ST group, a significant increase in test duration was obtained (respectively: 9.21 ± 2.02 vs 11.24 ± 1.26 min; *p* < 0.001 and 9.41 ± 0.39 vs 10.91 ± 2.22 min; *p* < 0.001). The extension of its duration is an effect that confirms the high effectiveness of the applied rehabilitation models and proves the expected increase in exercise tolerance. Another indicator showing the improvement of the physical capacity of patients, which significantly improved in both studied groups, is metabolic equivalents (MET) (IC group—9.16 ± 1.30 vs 10.73 ± 1.23, *p* = 0.006; ST group—8.65 ± 0.25 vs 9.86 ± 1.12; *p* = 0.002).

The myocardial oxygen demand depends on the heart rate, the tension of the left ventricular wall and the contractility of the heart muscle. According to Myers et al. [19] peak exercise capacity measured in MET is the strongest prognostic factor for the risk of death both among healthy people and those with cardiovascular diseases, including those after myocardial infarction. A favorable increase in the value of MET after the completion of the second stage cardiac rehabilitation program was also observed in the retrospective analysis of the results of 10,671 patients, regardless of their initial level of exercise tolerance [20]. A similar effect associated with the increase in MET was observed in the evaluation of the effects of hybrid rehabilitation of 125 patients with heart failure [21]. The increase in MET energy expenditure at a similar level was also demonstrated in the studies by Nowak et al. [4] and Grabara et al. [6], who also assessed the effectiveness of alternative training methods in cardiac rehabilitation of patients after myocardial infarction. Maximum oxygen uptake (VO_2_max), also referred to as the body's aerobic capacity, is a real measure of exercise tolerance and, at the same time, an indicator of the cardiovascular system efficiency [4]. It provides objective information about the clinical condition and factors limiting the possibilities of a cardiac patient [22]. A maximum oxygen intake of 10 mL/kg/min represents severe heart failure. The minimum level of physical activity assessed via VO_2_max is 40 mL/kg/min. For a person with a sedentary lifestyle, VO_2_max is approximately 30 ml/kg/min. The results of our research showed an increase (about 8%) in the VO_2_max value in both groups (IC group 37.27 ± 3.23 vs 39.10 ± 3.17 ml/kg/min; *p* < 0.001, ST group: 36.89 ± 6.22 vs 38.76 ± 3.44 ml/kg/min; *p* < 0.001). Endurance training increases oxygen uptake. It is the result of an increased capillary arteriovenous difference and an increase in cardiac output [23]. In the group of healthy people, the increase in VO_2_ max by 8–15% is the result of properly planned training. The similar increase achieved in our research proves that a properly planned rehabilitation program carried out in a continuous and systematic manner significantly improved the level of physical fitness of patients. This is also confirmed in the research by other authors [24–27]. The intergroup analysis did not show any statistically significant differences in the results of the research in terms of individual indicators.

### Echocardiographic test

The result of myocardial infarction is impairment of the mechanical function of the myocardium and progressive structural changes in the myocardium (called remodeling), which affect all parts of the cardiovascular system equally [28]. Altered hemodynamic conditions (for instance reduction of left ventricular stroke volume—LVSV, enhancement of LVEDV) and increased activity of the renin–angiotensin–aldosterone and catecholamines consistently contribute to impaired diastolic function of the heart, thus affecting the systolic function with a reduction in LVEF in total. First of all, the activity of aldosterone leads to the replacement of contractile muscle tissue with an excess of connective tissue with a predominance of collagen, which initially is an adaptive response, and later may take the form of pathological heart failure [29]. In addition to stimulating the renin–angiotensin–aldosterone system, diabetes mellitus, anterior infarction and its extensive early spread, and persistent occlusion of the intra-infarct artery exacerbate adverse myocardial remodeling. Reconstruction, and, more specifically, enlargement of the left ventricle silhouette may be a significant prognostic factor; therefore the assessment of its dimensions and functions should be routinely performed in most cardiological diseases. The study assessed the indicators of the left ventricle of the heart muscle. There were statistically insignificant increases in mean values of LVEDD, LVESD, LVESV, LVEDV, LVEF, LVMI, LVMI and a slight decrease in LVSV in both rehabilitated groups, which indicates a positive rehabilitation effect. It should be emphasized, however, that it is still ambiguous to determine the impact of physical activity (primarily of the endurance type) on the post-infarction structure and functions of the left ventricle [30]. The causes of this problem may include differences in the methodology of research carried out by different authors. The differences in the obtained results may be influenced by factors such as selection of the population, the extent of myocardial infarction, the age of the respondents, the period covered by the observation, measurement techniques and a combination of any of the above-mentioned factors. Similar conclusions were reached by Gates et al. [31], Belardinelli et al. [32] and Nowak et al. [33]. With the exception of the ejection fraction of the left ventricle, they did not observe any significant changes in the diastolic function of the left ventricle under the influence of training, even in relation to physically more or less active patients.

In conclusion, the influence of physical training on the heart has not been clearly explained. Most studies, including ours, failed to demonstrate a significant effect of physical training on the morphological and functional parameters of the left ventricle, or it was found that physical activity only slightly improved them. As in the case of the exercise test, the intergroup analysis showed no statistically significant differences.

### Examination of the lipid profile

Increased levels of total cholesterol and triglycerides are factors in the formation of atherosclerotic lesions in the coronary, cerebral and peripheral vessels. Their concentrations in blood serum are determined heredity, but a significant role in lowering the levels is attributed to lifestyle elements (environmental factors), such as a proper diet and systematic physical activity [34, 35]. Scientific reports confirm the beneficial effect of physical activity on the lipid profile, although it concerns longer observations, e.g. 6 months [32, 33]. In the case of observations that cover a short period of time, the changes are not statistically significant, which was also the case in our own research. It is also difficult to say whether the reason for the changes observed is the rehabilitation program or the effect of statins. Comparing the results of the tests before and after the start of rehabilitation, the level of the analyzed lipids in both cases was within the normal range, which may be even more indicative of the earlier undertaking of pharmacological treatment.

## Limitation

A limitation of the study was the inclusion of only a single group of patients with a high level of physical capacity ≥ 7 MET or ≥ 100 W. However, the results, which confirmed the effectiveness of indoor cycling training included in the cardiac rehabilitation program of patients after myocardial infarction, certainly warrant additional studies in this field, which will assess patient groups with lower physical capacity than those included in our study. A second limitation is the inclusion of the male study participants only. This was a select group of patients (see exclusion criteria) and may not be representative of a general cardiac rehabilitation population. Studies involving a large group of participants of both sexes with various levels of physical capacity and clinical status are needed.

In the future, research should also be undertaken to determine which of the indoor cycling techniques and profile is the most appropriate for cardiac patients.

## Practical recommendation

Indoor cycling training is a form of training that has many features in common with traditional endurance training used in cardiac rehabilitation. The obtained results also showed similarity in terms of changes in exercise tolerance, left ventricular hemodynamics and lipid profile. Therefore, it may be a more interesting and attractive alternative to traditional endurance training in patients after a heart attack.

## Conclusions

1. Both indoor cycling training and standard training have a similar effect on the improvement of exercise tolerance, change of hemodynamic indicators of the left ventricle and the lipid profile in patients after a heart attack.
2. Indoor cycling training can be an alternative to standard endurance training in cardiac rehabilitation.

## Data Availability

Not applicable.
